# Reviewed article: Curado DSP, Silva EV. Drugs incorporated into the Brazilian Unified Health System: documentary analysis of methodological decisions in budget impact analyses between 2012 and 2024. Epidemiol Serv Saude. 2025:34;e20250122.

**DOI:** 10.1590/S2237-96222025v34e20250122.b

**Published:** 2025-09-08

**Authors:** Fernanda Fávero Alberti

**Affiliations:** UFRGS, Medicina Social - Programa de Pós Graduação em Epidemiologia

**Table:** 

Rating	Excellent	Good	Average	Below average	Poor
Interest	✓				
Quality	✓				
Originality	✓				
Overall	✓				

**Table:** 

Questionnaire	Yes	No	Not applicable
Does the manuscript contain new and significant information to justify publication?	✓		
Does the Abstract (Summary) clearly and accurately describe the content of the article?	✓		
Is the problem significant and concisely stated?	✓		
Are the methods described comprehensively?	✓		
Are the interpretations and conclusions justified by the results?	✓		
Is adequate reference made to other work in the field?	✓		

## Manuscript Structure

Length of article is: Adequate

Number of tables is: Adequate

Number of figures is: Adequate

Please state any conflict(s) of interest that you have in relation to the review of this paper (state “none” if this is not applicable): None

## Comments

O artigo é descritivo e sistematiza as decisões metodológicas adotadas nos estudos de impacto orçamentário, um dos principais critérios para a incorporação de tecnologias em saúde. A escolha de uma análise documental de relatórios oficiais fortalece a confiabilidade dos dados. Além disso, o artigo segue a ferramenta RECORD, garantindo rigor na condução e no relato da pesquisa. Como implicações para o SUS, o estudo reforça a necessidade de maior padronização e transparência na condução das AIO no Brasil. Também destaca a importância de revisar as diretrizes metodológicas da Conitec para garantir que todas as incorporações sigam critérios robustos e alinhados com a sustentabilidade do SUS. 

